# Special Issue “Computer Aided Diagnosis Sensors”

**DOI:** 10.3390/s22208052

**Published:** 2022-10-21

**Authors:** Ayman El-Baz, Guruprasad A. Giridharan, Ahmed Shalaby, Ali H. Mahmoud, Mohammed Ghazal

**Affiliations:** 1 Bioengineering Department, University of Louisville, Louisville, KY 40292, USA; 2 Electrical, Computer, and Biomedical Engineering Department, Abu Dhabi University, Abu Dhabi 59911, United Arab Emirates

## 1. Introduction

Sensors used to diagnose, monitor or treat diseases in the medical domain are known as medical sensors. There are several types of medical sensors that can be utilized for various applications, such as temperature probes; force sensors; pressure sensors; oximeters; electrocardiogram sensors which measure the electrical activity of the heart; heart rate sensors; electroencephalogram sensors, which measure the electrical activity of the brain; electromyogram sensors that record electrical activity produced by skeletal muscles; and respiration-rate sensors that count how many times the chest rises in a minute. The output of these sensors used to be interpreted by humans, which was time consuming and tedious; however, interpretation became easy with the advance in artificial intelligence (AI) techniques and the integration of the sensor outputs into computer-aided diagnostic (CAD) systems.

This Special Issue has accepted 34 papers that present some of the state-of-the-art AI approaches used to diagnose different diseases and disorders based on the data collected from different medical sensors. This contributes towards achieving our goal, which is to develop comprehensive and automated computer-aided diagnosis tools by focusing on the different machine learning algorithms that can be used for this purpose as well as novel applications in the medical field.

## 2. Overview of Contribution

Fraiwan and Faouri [1] used deep transfer learning for the automatic detection and classification of skin cancer. Al Mudawi and Alazeb [2] presented an astute way to predict cervical cancer with machine learning (ML) algorithms. AlSaeed and Omar [3] proposed a pre-trained convolutional neural network (CNN) deep learning model (ResNet50) as an automatic feature extraction method for diagnosing Alzheimer’s disease from magnetic resonance imaging (MRI). Yasser et al. [4] described a novel framework that can detect diabetic retinopathy (DR) from optical coherence tomography angiography (OCTA) based on capturing the appearance and morphological markers of the retinal vascular system. Holubiac et al. [5] discussed the effect of a strength-training protocol on bone mineral density for postmenopausal women with osteopenia/osteoporosis assessed by dual-energy X-ray absorptiometry (DEXA). Ayyad et al. [6] proposed a new framework for the precise identification of prostatic adenocarcinoma from two imaging modalities.

Tariq et al. [7] proposed a novel feature-based fusion network called FDC-FS for classifying heart and lung sounds. ElNakieb et al. [8] provided a thorough study of implementing feature engineering tools to find discriminant insights from brain imaging of white-matter connectivity and using a machine learning framework for the accurate classification of autistic individuals. Diab et al. [9] presented a brain strategy algorithm for multiple-object tracking based on merging semantic attributes and appearance features. Fraiwan et al. [10] presented a non-contact spirometry using a mobile thermal camera and AI regression. Ramesh et al. [11] proposed the design and implementation of an explainable deep learning 1D-CNN model for use in smart healthcare systems with general-purpose devices such as smart wearables and smartphones. Liang et al. [12] developed a new flow sensor-based suction index from a measured pump flow (SIMPF) control strategy for rotary left ventricular assist devices (LVADs) to provide adequate cardiac output and prevent left ventricle (LV) suction.

Al-Mohannadi et al. [13] proposed a deep-learning-based approach to apply semantic segmentation for the intima-media complex (IMC) and to calculate the cIMT measurement. Alshboul and Fraiwan [14] developed an algorithm to count the number of chews in eating video recordings using discrete wavelet decomposition and low pass filtration. Hammouda et al. [15] introduced a deep learning-based CAD system to classify the grade groups (GG) system using digitized prostate biopsy specimens (PBSs) using pyramidal CNN, with patch- and pixel-wise classifications. Ahmad et al. [16] provided proof-of-principle for an optical-based, quick, simple, and sensitive screening technology for the detection of SARS-CoV-2, utilizing antigen-antibody binding interactions. Fournelle et al. [17] developed a new mobile ultrasound device for long-term and automated bladder monitoring without user interaction consisting of 32 transmit and receive electronic components as well as a 32-element, phased array, 3 MHz transducer. Khasawneh et al. [18] customized and pre-trained deep learning models based on convolutional neural networks were used to detect pneumonia caused by COVID-19 respiratory complications. Al Ahmad et al. [19] presented a novel immunophenotyping technique using electrical characterization to differentiate between the following two most important cell types of the innate immune system: dendritic cells (DCs) and macrophages (MACs).

Haweel et al. [20] proposed a novel CAD framework to classify 50 autism spectrum disorder (ASD) and 50 typically developed toddlers with the adoption of CNN deep networks. The CAD system includes both local and global diagnosis in a response to speech task. Sharafeldeen et al. [21] presented a new segmentation technique for delineating the lung region in 3D computed tomography (CT) images. To accurately model the distribution of Hounsfield scale values within both chest and lung regions, a new probabilistic model is developed that depends on a linear combination of Gaussians (LCG). Haggag et al. [22] proposed a novel framework for the automatic quantification of the vitreous on optical coherence tomography (OCT) with application for use in the grading of vitreous inflammation. The proposed pipeline consists of two stages, vitreous region segmentation followed by a neural network classifier. In the first stage, the vitreous region is automatically segmented using a U-net CNN (U-CNN). El-Gamal et al. [23] developed a personalized, cortical region-based CAD system that helps visualize the severity of Alzheimer’s disease (AD) in different local brain regions. Shehata et al. [24] developed a comprehensive CAD system for the early assessment of renal cancer tumors. The CAD system identifies and integrates the optimal discriminating morphological, textural, and functional features that best describe the malignancy status of a given renal tumor. Alwateer et al. [25] introduced a novel approach for processing healthcare data and predicting useful information with minimum computational cost, using a hybrid algorithm that consists of two phases.

Wagner et al. [26] compared a medical-grade electrocardiography (ECG) system with an ECG sensor of the low-cost DiY (Do-it-Yourself) hardware toolkit BITalino. Their results showed that the BITalino system can be considered as an equivalent recording device for stationary ECG recordings in psychophysiological experiments. Naglah et al. [27] proposed a novel multimodal MRI-based CAD system that differentiates malignant from benign thyroid nodules, based on a novel CNN-based texture learning architecture. Alyoubi et al. [28] proposed a screening system for DR fundus image classification and lesions Localization to help ophthalmologists determine the patients’ DR stage. Abdelmaksoud et al. [29] developed a CAD system that detects and identifies prostate cancer from diffusion-weighted imaging (DWI). The identification of prostate cancer was achieved using two previously trained CNN models (AlexNet and VGGNet) that were fed with the estimated ADC maps of the segmented prostate regions. Jo et al. [30] introduced a novel customized optical imaging system for human conjunctiva with deep learning-based segmentation and motion correction. The image segmentation process was performed by the Attention-UNet structure to achieve high-performance segmentation results in conjunctiva images with motion blur.

Dghim et al. [31] evaluated two different strategies of the automatic detection and recognition of Nosema cells from microscopic images and achieved the identification of a robust and successful methodology for automated identification and recognition of Nosema cells versus the other existing objects in the same microscopic images. Hasnul et al. [32] presented a review on emotion recognition research that adopted electrocardiograms as a unimodal approach as well as part of a multimodal approach for emotion-recognition systems. Ayyad et al. [33] presented a literature review of the use of histopathology images and its challenges in detecting prostate cancer, studying different steps of the histopathology image analysis methodology. Santos et al. [34] proposed a new approach based on image-processing techniques, data augmentation, transfer learning, and deep neural networks to assist in the medical diagnosis of fundus lesions.

We express our heartfelt thanks to all the authors for their contributions. We also thank the reviewers for volunteering their time to provide insightful comments and criticism on the submissions. Finally, we appreciate the support of the Editorial Board and Editorial Office of *MDPI Sensors* for making this Special Issue possible.

## Data Availability

Not applicable.
